# From precision interventions to precision health

**DOI:** 10.1038/s41467-025-60395-z

**Published:** 2025-05-30

**Authors:** Nicholas J. Schork, Laura H. Goetz

**Affiliations:** 1 The Translational Genomics Research Institute, a part of the City of Hope National Medical Center, Phoenix, AZ (NJS, LHG) USA; 2 The City of Hope National Medical Center, Duarte, CA (NJS) USA; 3 University of California San Diego, La Jolla, CA (NJS) USA; 4 Scripps Research, La Jolla, CA (NJS) USA; 5 Net.bio, Santa Monica, CA (NJS, LHG) USA

**Keywords:** Health policy, Translational research

## Abstract

Precision medicines, or those medicines that are tailored to individual genetic, molecular, physiologic, behavioral, and/or exposure profiles, are being developed at a rapid pace. However, just how precise these interventions are in terms of their mechanisms of action (MOAs), clinical effects, and utility in different individuals, are hard anticipate with current preclinical research and clinical trials strategies. To understand how various genes, processes, organs, clinical phenotypes, etc. may be impacted by an intervention, as well as how many people might benefit from it, appropriate data on living human beings needs to be collected as part of built-for-purpose clinical trials.

## Abstract (web summary)

We consider the shift from broad, population-level medicine to ultra-precise, individualized medicine using emerging therapeutic constructs, AI, deep phenotyping, and individual-centered trials to optimize health across diverse individual genetic and physiological profiles.

## Introduction

The availability of cost-effective high-throughput biomedical assays, such as DNA sequencing, proteomics, and metabolomics platforms, has enabled research designed to identify and characterize molecular processes that, when perturbed, underly diseases. The results of this research are unequivocal and suggest that most rare and common chronic conditions are multifactorial and exhibit etiologic heterogeneity. This heterogeneity raises important clinical and healthcare issues, such as the best way to make and categorize disease diagnoses, the best strategies for optimizing and deploying prevention and treatment practices, and the most appropriate ways to monitor intervention responses and overall health trajectories of individuals. Efforts to address these issues have led to the realization that for many conditions there is a need to precisely tailor (i.e., ‘personalize’ or ‘individualize’) prevention, treatment, and health monitoring strategies to an individual’s nuanced or unique genetic, molecular, physiologic, behavioral, and exposure profiles. How best to match, discover, and/or develop precision interventions for individual patients are open questions, as recent experience suggests^1–3^.

There are two important distinctions in the practice of precision medicine. The first distinction is between interventions that only benefit a single individual or subgroup of individuals vs. those that have a more ubiquitous effect. The second distinction is between health interventions that are meant to affect a very specific phenotype or circumscribed set of symptoms based on their mechanisms of action (MOA) vs. those that have a broad effect on the body, potentially enhancing or treating multiple phenotypes and symptoms. The definition of precision medicine emphasizes the need for crafting interventions that are truly tailored to an individual, implying that those interventions may need to have a limited clinical or symptom effect, because that is all that is needed for a particular individual. In this light, it is easy to accept aspects of precision medicine as inevitable with or without the use of interventions that have actually been tailored to specific individuals, since the combination of interventions any one individual may need over a lifetime would distinguish the unique health needs of that individual.

These two distinctions can be blurry, as experience suggests with the rapid development of many emerging precision health interventions and intervention types, such as Antisense Oligonucleotides (ASOs)^4^, neo-antigen targeting cytotoxic T-cells^5^, targeted electroceuticals^6^, probiotic mixtures^7^, and digital therapeutics^8^. In addition, since the goal of modern medicine is to restore or maintain health, it may be that in a precision medicine era a physician will draw on different interventions that are personalized or focused in their actions, and others with broader effects, over peoples’ lives. We ultimately promote the view that the two distinctions expose questions that are almost entirely empirical in nature. Therefore, we need strategies to generate appropriate insights into how best to provide precision preventive and treatment interventions to individuals. We provide a discussion, a few frameworks, and a vision of necessary infrastructure for pursuing precision medicine and health research and practice, arguing that precision interventions, even those that are patient-specific and ‘ultra-precise,’ require serious experimental evaluation to reveal their benefits and effects on the human body. This is especially the case in the light of growing insights into the highly complex and variable genetic and epigenetic determinants of diseases. We contrast interventions with a very circumscribed effect, such as some pain medications and antibiotics, with those that are meant to have a very broad effect, such as the emerging class of drugs known as ‘geroprotectors’ which impact multiple biophysiologic systems relevant to age-related diseases^9,10^. We also argue for the combined use of Artificial-Intelligence (AI)-based population-scale prediction modeling and aggregated, deep phenotyping, single case experimental designs (SCEDs)^11^—which encompass N-of-1 clinical trials but are not necessarily equivalent to them^12–14^—to pursue relevant studies, assuming the right researcher mindset and infrastructure can be generated.

## Challenges in precision medicine and health and potential solutions

### The breadth of an intervention’s effects

Consider the simple 2 × 2 categorization of interventions provided in Table 1, based on how patient-specific their MOAs are as well as how broad their molecular, physiological and clinical effects are in individuals receiving those interventions. As noted, interventions could be very patient specific in terms of their targets and hypothesized MOA but have broad pleiotropic effects of clinical significance. For example, an antisense oligonucleotide (ASO) designed to block part of a gene harboring a unique mutation possessed by an individual with a debilitating syndromic condition could alleviate many symptoms^4^. Many intervention targets (e.g., the GLP-1 receptor, or the gene target of an ASO) are parts of broader molecular and physiological networks (e.g., DNA repair, tissue formation processes, etc.)^15–17^. As such any downstream physiological and clinical impacts on those processes that may arise when the target is perturbed via an intervention may be hard to predict without appropriate human in vivo data. Other interventions with an individual-specific target could have a very circumscribed effect (e.g., patient-specific neoantigen targeting cytotoxic T-cells that kill only the neoantigen-bearing cells -- although cancer can affect the whole body^5^). Interventions could also be designed to have (or, importantly, only hypothesized to have) a very circumscribed clinical effect but modulate or target a very general, non-individual-specific mechanism, such as many pain medications (e.g., acetaminophen). This is often desired, since off-target effects could be, and likely are, negative, so having a non-individual-specific target but exhibiting little off-target effects could maximize the population benefit of the intervention. However, an intervention with limited clinical effects may not be a total treatment solution for an individual with a pathology that would require something beyond that intervention’s MOA and clinical effects. Other interventions, such as vitamins, especially in the setting of patients with vitamin deficiencies, may also target a very general, non-patient-specific mechanism and yet also have pleiotropic and broad physiological and clinical effects.

**Table 1:** A simplistic but paradigmatic categorization of interventions with example interventions in each category

	Breadth of Activity and Effects	
Target and mechanism of action (MOA)	Focused	Broad (Pleiotropic)
**Patient-specific**	Engineered neo-antigen targeting cytotoxic T cells	Some antisense oligonucleotides (ASOs)
**General mechanism**	Antivirals; some pain medications	Vitamins; GLP-1 Receptor Agonists; Geroprotectors

Whereas the criteria differentiating interventions with patient-specific versus general targets is in some cases straightforward (e.g., ‘does this patient have the stretch of DNA sequence targeted by an ASO in their genome?’^18^), this is not the case for all interventions billed as ‘precise.’ Some interventions thought to target, e.g., unique mutant proteins or mutant-bearing DNA or RNA sequences, actually exhibit activity among, and may benefit, individuals who have different mutations^19–21^. Differentiating interventions with limited physiological and clinical effects (i.e., those that are not pleiotropic) from those with broad effects is difficult in practice for many reasons. First, despite great sophistication in molecular pharmacology research and resulting insights into, e.g., target selectivity involving different receptor conformations, target binding strengths, etc. to ensure an appropriate MOA^22^, human physiology involves so many redundancy, feedback, and interactive processes and mechanisms^15–17^ that opportunities for unique combinations of perturbations, genetic or otherwise, arise and ultimately impact an intervention’s downstream physiological and clinical effects for any individual. Such activity is not directly interrogated in most cellular constructs used in early-stage drug development.

Second, the effects of an intervention may be ‘primary’ and associated with the intervention’s MOA, or ‘secondary’ and associated with the downstream consequences of a particular gene or process targeted by an intervention (e.g., weight loss induced by various interventions could lead to improvements in blood pressure, cholesterol and hyperglycemia). Importantly, the *broader* clinical and physiological effects, positive or negative, of interventions may never be explored given that many interventions are developed to pass regulatory approval processes that require associating interventions with a singular ‘primary endpoint’ in the form of a symptom or condition. Consider the most promising class of weight management medications, the glucagon-like peptide-1 (GLP-1) receptor agonists and dual GLP-1/GIP (gastric inhibitory polypeptide) receptor agonists. These medications initially were limited in their use for treating Type 2 Diabetes Mellitus but are now, after empirical studies, thought to have broad effects on a wide range of phenotypes and conditions such as obesity, cardiovascular disease, obstructive sleep apnea, renal function, nicotine and alcohol cravings, and neurodegenerative diseases^23–25^.

Another example of interventions defined by their broad pleiotropic and clinically relevant effects is the emerging class of interventions known as ‘geroprotectors,’ that act on many—maybe all—age-related diseases^10,26^. This raises serious questions about how geroprotector efficacy can be proven in appropriately designed clinical trials that do not focus exclusively on a single endpoint, disease, or primary outcome^10,13,27^. Yet another example involves recent developments to create implantable drug delivery systems that could control the release of multiple medications as needed, which is reminiscent of the concept of a ‘polypill’^28,29^.

The point of these distinctions is not to offer a definitive (and perhaps illusory) categorization of efforts to develop interventions, but rather to point out that appropriate research could be compromised by many current conflicting foci, such as on basic, pre-clinical science and not translational or clinical science among academics, or regulatory emphases on primary endpoints in pivotal trials that limit whole body insights among pharmaceutical and biotech companies, as well as stakeholder – including patient – demands that conflict with public funding and commercial investments. Ultimately knowing just how interventions affect different individuals from whole body perspectives to improve their health trajectories are complex issues, and few biomedical science initiatives consider them^13^.

### Emerging ultra precision interventions

**Fig. 1:**
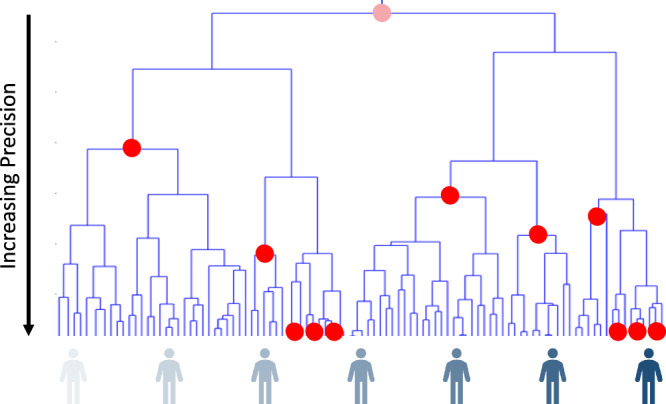
Individual similarities in profiles guides precision medicine. Graphical depiction of different sized groups of individuals who will benefit from specific interventions based on the similarity of those individuals with respect to their genetic, molecular, physiologic, behavioral and exposure profiles. The dendrogram reflects the similarity of the patients. The gray figures underneath the dendrogram are individuals with varying levels of a particular biomarker (darker = higher levels). The red dots reflect different interventions such that the branches underneath them indicate individuals who would benefit from those interventions (e.g., males vs. females; those with a genetic profile vs. those without, etc.). The pink dot at the top reflects an intervention that would benefit everyone, and the red dots at the lowest points in the figure reflect ultra-precise patient-specific interventions. Adapted from^13^.

There are many interventions that have been tailored or personalized in some way^30^. Many cancer therapies, for example, have MOAs that are known to counteract the effects of specific tumor mutations and are prescribed with this in mind^31,32^. However, some interventions are more ‘precise’ in their targets and MOAs than others. For example, following Fig. 1^13^, one could distinguish ‘one-size-fits-all’ medicines which work in all individuals, ‘stratified medicines’ in which a few markers (e.g., sex and age) might be used to determine which individuals get certain treatments, ‘precision medicines’ in which more markers (e.g., based on genetic or microbiome profiles) are used to identify smaller subsets of individuals, each getting a specific intervention, and ‘ultra-precise’ or ‘individualized medicines’ in which each patient receives a unique intervention^33^. Identifying the markers that can be used reliably to determine who should be provided which treatments is a major goal of modern biomedical research^34,35^. Thus, given the different motivations for developing interventions and the current regulatory landscape, many questions about the utility of interventions in the population at large are empirical in nature and simply require further study to resolve.

There are several recent interventions that have been designed to be *ultra*-precise in their design, targets, and MOAs and a few have had precedent-setting, yet limited and tempered, success that suggest their clinical potential^36,37^. Many such interventions are being developed for rare diseases^38,39^. These interventions often focus on genomic phenomena such as rare germline mutations and their immediate molecular pathophysiologic consequences^40^, somatic cell replication-acquired mutations contributing to tumorigenesis, and viral and bacterial pathogens with unique genomic sequences. They also exploit a wide variety of therapeutic constructs, such as antisense oligonucleotides (ASOs)^18,36,41–43^ (see Table 2). However, not all ultra-precise interventions are designed to treat individuals with rare conditions. For example, highly patient-specific, tailored interventions have been developed to treat depression by using, e.g., imaging techniques to identify and then target key focal brain regions in an affected individual with electrotherapy^44^. Other patient-tailored interventions that are designed to address common chronic conditions include, for example, individualized nutritional plans or probiotics for Inflammatory Bowel Disease, metabolic syndrome, and cardiometabolic conditions^7^, just-in-time adaptive interventions (JITAIs) in behavioral health^45^, and individualized digital therapeutics for the treatment of addictions^8^.

**Table 2:** Examples of ultra-precision (single individual) interventions in which the intervention is designed to target likely patient-specific pathogenic mechanisms after a thorough examination

Intervention constructs	Example conditions	Example references
ASOs	-Batten’s disease -neuronal ceroid lipofuscinosis 7 (CLN 7) -Timothy Syndrome	^1,18,36,120^
Gene therapies	-hereditary spastic paraplegia type 50 - neuronopathic mucopolysaccharidosis type II (Hunter syndrome) -Metachromatic Leukodystrophy Wiskot-Aldrich Syndrome	^121–124^
CRISPR	-rare neurodegenerative diseases	^125^
RNA-Editing	-inherited retinal diseases (e.g., Stargardt Disease) -alpha-1 antitrypsin deficiency	^3,126^
CAR-T and cytotoxic T cells	Hematologic malignancies, melanoma	^5,127,128^
Probiotic mixtures	Cardiometabolic disease, metabolic syndrome, inflammatory bowel disease,	^7^
Imaging and electroceuticals	Major Depressive Disorder	^6^
Tailored digital therapeutics	Opioid Addiction	^8^

Three important aspects of the development and use of ultra-precise interventions, whether designed for individuals with rare conditions or not, should be emphasized. First, such interventions can and should be vetted at the population level as well as the individual level by asking if the broader ‘platform’ behind the ultra-precise interventions has utility relative to other intervention types. Thus, studies should be pursued that address questions such as ‘do individuals receiving ultra-precise ASOs do better than individuals who receive alternative (‘one-size-fits-all’) interventions or standard of care?’^33^. Second, ultra-precise interventions require additional items for their deployment and use beyond the actual interventions themselves (e.g. companion diagnostics, genomic profiling, imaging protocols, etc.). Population surveys focusing on the frequency with which relevant targeted phenomena occur could reveal potential disparities in needs or use^46^. Third, better characterization methods for screening, e.g., drugs and natural products using individual-derived cellular constructs such as organoids, induced neurons, or individual-specific lab-on-a-chip constructs, can yield insights into MOAs of interventions that might have benefit when provided to a specific individual^47,48^.

Another important question is how ultra-precision interventions, given their MOAs and the breadth of their effects, fit into the bigger picture of improving and sustaining an individual’s health over their lifetime. For example, in the context of the distinction about how broad the clinically relevant health effects of an intervention might be, an ultra-precise intervention may not be adequate to improve the individual’s health overall. Consider the fact that, despite the heroic efforts of, and major medical precedents set by, a team developing a patient-specific ASO to treat a young girl with Batten disease, the young girl died^36,49^. This could have been due to the intervention not overcoming the girl’s pathologically remodeled system, or due to an inability of the ASO to sustain its benefits over time, and indicates the need for even better characterizations of an intervention’s effect, to the degree relevant studies are logistically feasible, financially possible, and do not compromise or overburden study participants.

### Geroprotectors and the overall health of individuals

Although target selection and the design of interventions is often motivated by a desire to treat or intervene on a singular symptom, clinical manifestation, or an a priori defined set of clinical phenotypes associated with a particular disease, there is growing interest in developing health interventions with broad effects that fortify many health sustaining and curative processes (i.e., the right side of Table 1)^9,10^. Many believe that such ‘geroprotective’ interventions could work by slowing the aging rate, thereby curtailing age-related processes whose accumulating damage to the system leads to pathologies. Whether or not this achievable, and whether or not the current ways in which the aging rate is measured and analyzed (e.g., epigenetic or general biological clocks^50^) can reveal reliable geroprotective targets, are open questions^27,51^. Per Table 1, geroprotectors could in theory have very individual specific targets, but by definition must have pleiotropic and broad clinical effects. The design of clinical trials to vet such effects, along with the type of biomarkers and endpoints they would exploit, are growing research areas motivated by recent insights into the aging process^10,52,53^ (see, e.g., the XPRIZE Healthspan Competition^54^).

**Fig. 2:**
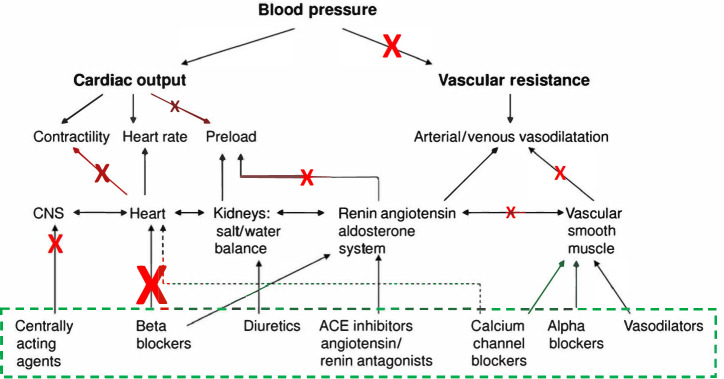
Pathways and processes involved in blood pressure regulation. Schematic representing factors involved in blood pressure (BP) regulation and perturbations, denoted by the red Xs, that may affect BP, and the different therapeutic targets for classes of antihypertensive depicted within the green dashed box. The large red X indicates a perturbation that affects heart rate that is a target for intervention for the class of drugs known as beta blockers. The smaller red Xs indicate other perturbations that could influence blood pressure regulation. Adapted from^69^.

The targets and MOAs of interventions that prevent disease and sustain health, as opposed to treat disease, are different from those meant to treat a specific disease or symptom, as are the trial designs to vet them. For example, some pharmacotherapies are designed to only attack cells or processes that appear *after* a pathogenic process has been initiated, thereby preventing progression to fulminant disease^55^. Preclinical models often cannot determine the effects of an intervention on human in vivo biology, in terms of not just efficacy and side effects, but also broader pleiotropic effects and phenomena that may thwart any benefit (Fig. 2 and Supplementary Fig. 1)^56,57^. This not only leads to many interventions failing in Phase II (i.e., efficacy studies)^58^, but also many interventions exhibiting poor benefit to non-benefit, as well as responder to non-responder, ratios after their approval^59^. As emphasized below, the solution is to pursue more studies that probe and expose human biology, the relationships between clinically relevant processes and phenotypes, and mechanisms that thwart intervention efficacy, without compromising ethical standards, participant burden, and logistical and financial practicalities^13,27,53,60^.

### Why interventions do not work ubiquitously

Health interventions, whether for improving public health generally or for rare debilitating diseases, rarely, if ever, work ubiquitously. The reasons for this are often underappreciated. Consider the following questions: would someone expect an intervention they are developing for a specific pathologic process to also benefit the *overall health* of an individual who is malnourished? has a debilitating rare condition? has been exposed to toxic substances? has been on treatments for many other conditions? or has been engaging in unhealthy behaviors? The answer is probably no, yet *all* humans exhibit these and other intervention effect-compromising issues to some degree. Beyond these, human genetic variation (genetic differences between individuals) generally impacts health and drug response in at least 4 ways: 1. Pharmacokinetic (PK) effects in which relevant, e.g., drug metabolizing enzymes (DMEs) and transporters are compromised by genetic perturbations^61^. 2. Pharmacodynamic (PD) effects, in which intervention targets, and possibly more downstream factors, that affect the activity of a drug are perturbed by a genetic variant (e.g., a genetic variant that upsets the target binding site of the drug)^61^; 3. The presence of a highly penetrant variant with systemic and debilitating effects (e.g., variants causing Batten disease or Cystic Fibrosis); note that ~5% of all humans harbor rare, likely health compromising variants^62^ and all individuals harbor some number of rare or unique functional variants whose clinical and physiological significance is unknown^63,64^; and 4. The physiologic burden induced by polygenic variants that all humans possess whose collective effects can be difficult, if not impossible, for an intervention to overcome^65^. Consider that an intervention’s specific MOA likely targets mechanisms upstream of many other genes, processes, or mechanisms, that contribute to the clinical phenotype of interest that could be perturbed^66^. This last issue was recently exposed in a large study of mice subjected to different forms of rigorous dietary restriction (DR) to explore the geroprotective effects of DR on lifespan^67^. Interestingly, not only did not all the mice benefit from DR, but genetic factors explained about 3 times more of the variation in lifespan than did DR^67,68^.

Of these four phenomena, the fourth is the most underappreciated. We have provided a schematic of relevant phenomena in Supplementary Fig. 1 which considers the influence of polygenic variation on the effects of an intervention. An intervention is often designed to modulate a specific factor (e.g., a gene, protein, process, etc.) that is appropriately upstream in the casual and feedback-laden hierarchy and network of factors impacting the clinical phenotype(s) or health state of interest^65^. The important question is whether the intervention, by focusing on the modulation of a specific factor known to be upstream of others, can overcome perturbations induced by, e.g., genetic (or epigenetic) variants, in factors or processes *downstream* of that target that also impact the clinical phenotype of interest. Consider Fig. 2^69^, which depicts various processes associated with blood pressure regulation. If an elevated heart rate appears to contribute to an individual’s high blood pressure, perhaps because of a deleterious variant in a relevant heart rate modulating gene, then that individual many benefit from a heart rate lowering drug (e.g., a beta-blocker). However, if the individual possesses genetic variants that contribute to stiffer blood vessels, impact catecholamine release, and affect centrally mediated control of the sympathetic nervous system, then the drug may not have as much overall benefit or improve individual health over time. Note that we accept that some genetic variants could be beneficial and enhance the functioning of a human body and its amenability to improvements induced by health interventions^70,71^.

Ultra-precision medicines—and in fact any intervention—can in theory overcome the first three of these genetic phenomena by, e.g., determining dose and companion interventions (e.g., nutritional interventions), but not necessarily the fourth, even in the context of rare diseases, since the number and complexity of intervention compromising factors may be large. In this light, one could ask what the ultimate goal of health care is: to reduce a very narrow set of symptoms with available interventions? combat all symptoms with a population-level orientation? or improve overall health of individuals, which may require complex considerations about each individual’s profile? What costs, scientifically and financially, would come with such efforts? In addition, although we have focused on germline genetic variations and mutations, somatic mutations, epigenetic phenomena, environmental exposures, certain behaviors, and complex interactions among any subset or all of them, could lead to perturbations that impact relevant processes or systems targeted by an intervention in an analogous manner to inherited variations. This could create an even greater complexity that health interventions would have to overcome^15–17^.

As emphasized throughout, the ability of any intervention to overcome other more subtle but collectively important perturbations beyond those targeted by that intervention is largely an empirical question, at least with respect to the current knowledge available to clinicians and researchers. This question is also relevant to the overall health of many individuals. We believe the fundamental contributor to this issue is a lack of appropriate studies in humans that could reveal relevant and crucial clinical insight into human biological phenomena associated with health interventions. Consider that most scientific experiments exploit a perturbation to a system of interest to see how elements in that system (e.g., individual genes, proteins, metabolites, processes like DNA repair, etc.) respond or interact. Purposely perturbing individual human beings for purely scientific gain or insight is unethical. However, when individuals subject themselves to health interventions of all sorts, whether in the context of an approved and regulated clinical trial or not, they are in effect perturbing the (or their) human system in some way (unless the intervention is inert). This fact provides the very context necessary to explore not only an intervention’s effects but the relationships between factors and processes that may be related to or correlated with the intervention’s hypothesized MOA^13^.

### Vetting interventions with precise MOAs or that act broadly

Standard clinical trials seek to determine if the average intervention response is meaningful (ignoring the fact that no one is actually ‘average’). This is consistent with both current regulatory practices and the way clinical medicine is pursued; i.e., putting a greater focus on treating disease within an organ system-based, singular symptom, and diagnosis framework as opposed to preventing disease, maintaining healthy states, and promoting whole body health^13^. This legacy way of vetting interventions downplays the limits of an intervention in certain individuals, or subgroups of individuals, and tends to ignore the breadth of the intervention’s effects on different health sustaining processes and clinical endpoints. Interestingly, over thirty years ago, Topol and Califf argued for balancing large population-based clinical trials that provide ‘definitive evidence about the mortality reduction afforded by a class of therapy so that broad changes in clinical practice can be justified’ with more focused trials that consider an intervention’s MOA and the breadth of its activity and clinical effects^72^.

Recognition of the limits of traditional approaches to clinical intervention development and clinical trial paradigms has created a need for more innovative approaches^37,73,74^. Also, the contemporary emphasis on precision medicine, especially with the emergence of ultra-precision interventions, is motivating the design of more individual-focused, whole body health trials^37,75^. This recognition is consistent with the results of, e.g., large-scale genome-wide association studies (GWAS), focused genetic studies^66,76–78^, and electronic medical record (EMR) and insurance claims database data mining studies exploring responsive subgroups to various health interventions^79^. It is also consistent with studies involving organoid and related constructs derived from specific individuals exploiting drug screening which have revealed interventions whose MOAs and effects appear to counteract the pathologies exhibited by those individuals^47,48,55^. A prevailing theme in modern molecular biology is the interconnectedness of various processes under genetic and epigenetic control that defy reductionist approaches to their characterization^16,17^. Efforts have long been underway to sort out which factors (e.g., transcripts, proteins, metabolites, etc.) and processes (e.g., DNA repair, primary metabolism, apoptosis, etc.) are correlated, and which are primarily or secondarily affected when other factors or processes are modified, either in contrived (i.e., experimental) or natural settings. Therefore, it makes sense to consider broader connections between factors and phenomena at the molecular, physiological, organ, subclinical, and clinical levels in the light of the *perturbations* or modifications (hopefully positive) of different molecular factors that are affected by health interventions. To do this effectively new types of studies must be pursued that focus on general human biology and individual whole-body responses to interventions. These studies will require enough data to be collected on each enrolled study participant (i.e., deep phenotyping over time) so that more compelling and statistically powerful inferences can be drawn about their responses to interventions. Longitudinal data are essential since cross-sectional studies cannot address issues about the stability (i.e., consistency over time), stationarity (i.e., lack of physiological adaptions that reset the response in unique ways), and level of equilibrium (i.e., how long it takes for the drug to have a lasting and consistent effect) of factors that mediate response^80^. These studies could be pursued at virtually all phases of intervention development with emphases on the following.

### Identifying responsive subpopulations

Studies seeking to identify prognostic biomarkers, or those predictive of response^81^, are most powerful when longitudinal data are collected. This provides insight into the overall health trajectory of individuals, irrespective of what interventions they may be provided^81^.

### Characterizing the breadth of intervention effects

To determine the breadth of either direct or indirect effects of an intervention on clinically meaningful endpoints, more data on those endpoints must be collected on individuals enrolled in a trial^13,82^. Strategies used in early phase PK and PD studies or therapeutic drug monitoring (TDM) studies can be adopted, such as measuring plasma levels or markers of activity of a drug or nutrient of interest. This will allow correlations and causal inferences to be drawn between, e.g., drug levels, molecular activity, and clinical endpoints^60^.

### Repurposing studies

If experience with an intervention during its development or post-approval suggests that the intervention shows benefit for a previously unanticipated endpoint, then efficient patient-focused studies can be pursued to verify this. Such studies could precede larger studies to assess population efficacy for the new endpoint^72,83^.

### Characterizing the length of time before clinically meaningful effects manifest

It is important to know how long it may take an intervention to have its effects, especially if the intervention is thought to have geroprotective properties. For example, an individual receiving an intervention may be in a vulnerable state (e.g., due to a heart condition, rapidly progressive cancer, or cognitive decline) and does not necessarily have time to wait for an intervention with a very slow MOA to induce its health effects. This problem is even more pronounced in individuals with multiple health compromising conditions (or competing risks). This is also relevant to the distinction between interventions that are disease-modifying (i.e., those that affect, remodel or reverse underlying pathogenetic processes) versus those that only affect specific symptoms^84^. Trials seeking to address this question must use appropriate biomarkers or measures of relevant pathological processes collected over extended periods of time.

### Identification of better response biomarkers and surrogate endpoints

Many disease phenotypes are difficult to measure over time (e.g., tumor shrinkage or tumor biopsy derived markers) or include morbidity and mortality endpoints for characterizing important aspects of the desired effects of an intervention. To combat this, ‘surrogate endpoints’ can be used that capture a drug’s effect but are highly correlated with the more complicated outcome of interest (e.g., cholesterol level as a surrogate for heart disease; progression free survival as surrogate for overall survival in cancer)^85^. Such studies would benefit from extensive longitudinal data collections to accommodate more reliable causal and mediation effect claims linking intervention activity to clinical outcomes^86^.

### Refining an intervention and its use

By focusing on longitudinal and deep phenotype data, aspects of an intervention’s effect could be revealed that indicate a need for an alternative target for the intervention, a refinement of a single intervention, or a combination of interventions. In relevant studies, the synergistic effects of combinations of interventions, and the biological basis and implications of important and perhaps deleterious drug/drug or drug/diet or exposure interactions, could be assessed in earnest^87,88^.

### Benefitting patients in real time

Many traditional population-based randomized controlled trials (RCTs) do not typically provide feedback to trial participants and rarely collect enough data on them to make meaningful claims about their health trajectory. However, individual-focused studies with deeper phenotyping and longitudinal monitoring are appropriate vehicles for engaging those individuals and learning about their unique health trajectories in real time, and could, as they are carried out, essentially provide a form of expanded care for them^89^. Such an expanded and unique focus on trial participant health can be a recruitment draw and an excellent vehicle for training physicians and translationally oriented biomedical researchers.

There are many designs that can be used to conduct studies emphasizing the themes described above but aggregated single case experimental designs (SCEDs) stand out as particularly useful^11^. SCEDs focus on data collections on individuals. Such studies encompass N-of-1 trials, in which repeated crossovers of an intervention and a comparator are exploited^12–14,90,91^, but can be broader in their conception and design. For example, interrupted time series designs are a SCED, which are used routinely in a wide variety of settings, and involve a baseline and intervention period with a number of statistical safeguards available for relevant analysIs^92^. Standard clinical trial techniques such as blinding, randomization and the use of washout periods could be exploited in such studies. Importantly, the aggregation of data from SCEDs – as long as there are common elements to them – could be used to assess predictive biomarkers, prognostic factors, patterns of response, inter-individual variation in response, and covariate effects, with excellent power if a sufficient number of studies are aggregated^93,94^. Individual SCED studies whose data and results are aggregated could also be designed to have equal representation from individuals with different covariate profiles, including sex and gender or ancestral backgrounds, thus ensuring generalizability of results of the aggregated analysis. In addition, aggregated SCEDs could be pursued sequentially making them very efficient in making go/no-go decisions about the future use of an intervention of interest^95^. The availability of reliable and effective health monitoring devices such as wearables, portable imaging, and EEG devices, as well dried blood spots technologies can enable and enhance relevant studies^96^ and potentially be used in actual clinical practice in settings where intervention equipoise exists^97,98^.

### Integrating precision medicine strategies at different scales

Massive electronic health record (EHR) databases^99^, large epidemiological cohort studies, such as the UK Biobank^100^ and the Danish Health Registry^101^, have motivated data mining initiatives that are meant to impact health care practices. These data mining initiatives have been strengthened by high-throughput, massively parallel assays such as DNA and RNA sequencing, proteomics, metabolomics platforms and imaging technologies, online survey instruments, environmental exposure monitors, and wireless health devices on the participants, such as the NHANES survey^102^ The goal of these initiatives is to generate data that can be aggregated and mined for patterns that might indicate individuals or groups of individuals likely to benefit from specific interventions. Many statistical techniques, including Artificial Intelligence (AI) and Machine Learning (ML) techniques, as well as statistical matching strategies and covariate balance techniques for assessing causality^103^, have been proposed for these purposes to the point where medical journals are preparing to endorse and critique them^104^. Strategies to leverage such ‘real world evidence (RWE)’ to make claims about intervention deployment and personalization is also getting attention from regulatory agencies, given an appreciation of the expense and impracticality of many prospective clinical trials^105^. However, RWE predictions and population-based study results may be imprecise and need further validation before they are translated into clinical practice. In fact, they could be coupled with studies characterizing individuals, such as those leveraging DNA or RNA sequencing to identify targets for intervention or, e.g. patient-derived cellular constructs for ex vivo drug testing, as well as a coordinated set of SCEDS to vet predictions.

**Fig. 3:**
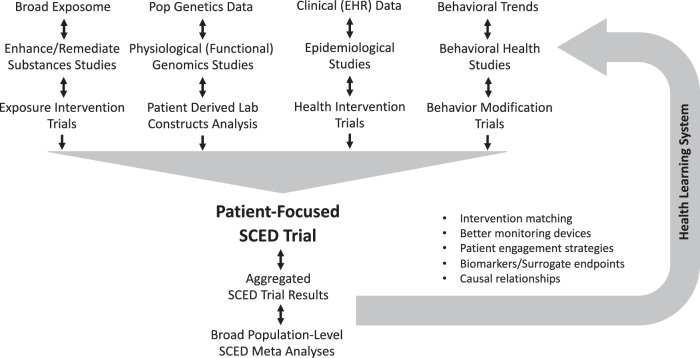
Clinical and health intervention learning systems. Broad levels at which precision health research is pursued and their potential contribution to, and benefit from, focused Single Case Experimental Design based trials  of individual patients. Each of the 4 columns in the upper part of Fig. 3 reflects a focus area in the literature seeking to identify factors, protocols, and/or strategies for treating individual patients differently based on their unique genetic, physiologic, exposure and behavioral profiles. The feedback between focused patient-oriented SCED studies and the use of population-based predictions can contribute to a true ‘Health Learning System’.

Figure 3 provides a schematic reflecting the proposed integration of RWE studies, patient-focused assessments, and patient-focused SCEDs to create what would amount to a ‘clinical and health learning (CHIL) system’^106^. Essentially, for any patient, available knowledge for determining which interventions might be appropriate for them at any point in time would be used to guide intervention choices. Some subset of these patients could undergo SCED-based trials to determine the effects of that intervention and vet the predictions. The results of these SCEDs could then be fed back to the knowledgebase exploited in the creation of the prediction models derived from the RWE and population studies, hopefully leading to improved predictions^33,107,108^. The necessary infrastructure to enable such integration and embark on relevant studies is crucial and would require unprecedented coordination, protocol sharing, the use of common data repositories, and sophisticated adaptive algorithms. However, initiatives such as US Clinical Translational Science Award (CTSA) program, made up of a network of 50+ sites, each equipped with personnel and resources to enable translational research, could provide necessary infrastructure^109^. In addition, a very recent and visionary review of the issues in setting up relevant clinical informatics resources could motivate rapid development of appropriate infrastructure^110^.

## Discussion

Recent biomedical research studies have produced new health monitoring technologies, different scientific visions, massive AI/ML-based data analyses, entrepreneurial and enterprise-level efforts meant to improve human health, and novel health interventions and strategies. However, current drug development, clinical, and public health initiatives face many challenges: the aging population, emerging infectious diseases, immigration and economic hardship leading to health disparities, health challenges associated with global climate changes, military conflicts, and general stress and anxiety due to political divisions. The key to overcoming these challenges will be harnessing and directing the interest and momentum behind contemporary biomedical science initiatives to understand human clinically relevant biology and intra- and inter-individual variation in intervention response in such a way as to meet the health needs of individuals. However, There are many serious impediments that will impact the pursuit of relevant studies as well as the infrastructure needed to conduct them. For example, the costs of individualized and precision clinical trials can be discouraging^18,111^ and getting stakeholder buy-in may be complicated given economic conditions and a potential desire to maintain the status quo^112^. Also, newer precision and ultra-precision medicines that require magistral (i.e., real time) production, as opposed to relying on creating and then stockpiling medicines for later distribution, require shifts in the supply chain that may be as disruptive as - and yet require - studies of the type proposed here^113^. For example, compounding with 3-D printing^114^, phage construction^115^, synthetic biology^116^, and the development of ASOs, gene therapies, and other intervention classes for rare disease generally^117^, will require a reset of the industry that may depend on, e.g., aggregated SCEDs to determine their viability and impact on the overall health of individuals^118^.

The use and deployment of ultra-precision interventions over the lifetime of individuals will in theory require assessments of those individuals to identify specific perturbations that underly their unique disease profiles and clinical manifestations (e.g., via DNA sequencing), followed by the pursuit of many clinical trials to vet interventions aimed at those perturbations. As emphasized, we believe that the integration and complementarity of population-level predictions, enhanced by the availability of ‘big data’ and AI and ML-based analysis tools, when coupled with the judicious but appropriately powered use of aggregated SCEDs, could lead to logistical and financial efficiencies. The requisite infrastructure (e.g., data repositories, computing facilities, clinics, etc.) and mind set (e.g., data sharing, credit sharing, etc.) will need to be in place^110^. In addition, the secondary benefits of the conduct of the proposed studies could motivate financial investment and patient participation in relevant initiatives. For example, the proposed SCEDs are excellent vehicles for training physicians and translational researchers since they combine exposure to new technologies (e.g., interventions, health monitoring devices, data analysis methods) in very practical patient-oriented settings^97,119^ They are also perfect for advancing patient engagement, dealing with sensitivities to participation in research, and vetting emerging products (e.g., new health biomarkers or monitoring devices) useful in a precision medicine era. Finally, as emphasized, because of the individual-focus of the trials and the deep phenotyping data emerging from them, one could learn about a participant’s condition during the conduct of the trial that might benefit them in real time.

Ultimately, major technical advances in the biomedical sciences, including the development of new interventions, both precise and not precise, circumscribed in their activity and not so circumscribed, and emerging health monitoring devices and assays of all sorts, raise questions about their impact and deployment. In fact, the very use and implementation of these interventions, monitoring devices, etc. are resulting in experiments on human biology whether acknowledged as such or not. How quickly and reliably the biomedical science community analyzes information from these experiments, enhances the collection of additional information from them, and takes advantage of the results, will determine how soon we can improve the health of everyone.

## Supplementary information

Supplementary Information
