# Supplementary material for: From precision interventions to precision health
Source: Nat Commun. 2025 May 30;16:5024. doi: 10.1038/s41467-025-60395-z (PMC12125267; doi:10.1038/s41467-025-60395-z)
Supplement: Supplementary file 1 — Supplementary Information [file 41467_2025_60395_MOESM1_ESM.pdf]

Supplementary Figure 1. Schematic depicting complexities down stream of intervention target engagement. Very basic model of factors involved in temporal processes triggered by the administration of a drug targeting a specific factor and the chain of events elicited by the modulation of the target that lead to clinically relevant health states.

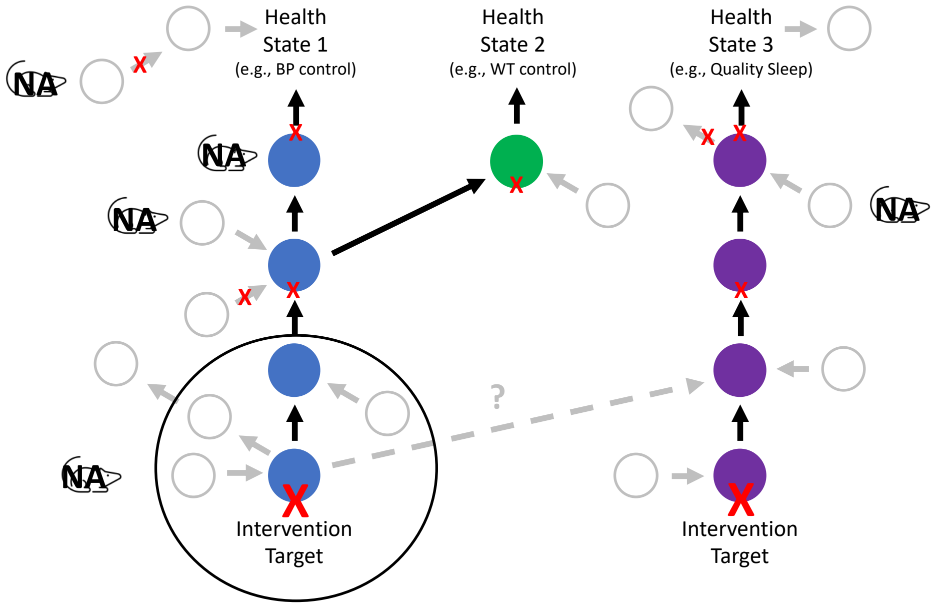

Key: Circles depict factors (e.g., genes, proteins, metabolites, cells, extracellular vesicles, etc.) and processes (e.g., fluid balance, blood pressure, tissue and organ function). Colored circles depict the main factors or processes in a particular pathway whose interconnections are reflected by arrows that lead from a factor targeted by an intervention to modifications in clinically relevant health states or phenotypes. Gray arrows and unfilled gray circles indicate other factors that impact those (colored) factors reflected in the main pathway. The large red Xs reflect perturbations that interventions are designed to overcome and correct. The smaller red Xs reflect perturbations (e.g., genetic variants or mutations) affecting factors downstream of the factor targeted by the intervention. The dashed line connects the mechanism of action (MOA) of the intervention affecting health state 1 and 2 to factors involved in modulating health state 3. Note that if there is no such connection, then modulating health state 1 will not affect health state 3, such that if health state 3 is compromised, the individual will not be healthy despite improvements in health states 1 and 2. The large black circle surrounding the intervention target for the blue pathway reflects limitations of the insights obtained from studies involving cellular constructs and the figures of mice with NA (Not Applicable) reflect biological differences between humans and mice that may compromise the use of mouse models to understand how an intervention impacts human biology.
